# Sinusoidal electromagnetic fields accelerate bone regeneration by boosting the multifunctionality of bone marrow mesenchymal stem cells

**DOI:** 10.1186/s13287-021-02302-z

**Published:** 2021-04-13

**Authors:** Weigang Li, Wenbin Liu, Wei Wang, Jiachen Wang, Tian Ma, Jingyuan Chen, Hua Wu, Chaoxu Liu

**Affiliations:** 1 Department of Orthopedics, Tongji Hospital, Tongji Medical College, Huazhong University of Science and Technology, Wuhan, 430030 Hubei China; 2 Department of Orthopedics, Xiangya Hospital of Central South University, Changsha, 410008 Hunan China; 3 Department of Hematology, Tongji Hospital, Tongji Medical College, Huazhong University of Science and Technology, Wuhan, 430030 Hubei China; 4 Department of Cardiothoracic Surgery, Tongji Hospital, Tongji Medical College, Huazhong University of Science and Technology, Wuhan, 430030 Hubei China

**Keywords:** Stem cells, Sinusoidal electromagnetic field, BMP receptors, Osteogenesis, Angiogenesis, Osteoimmunomodulation, Bone tissue engineering

## Abstract

**Background:**

The repair of critical-sized bone defects is always a challenging problem. Electromagnetic fields (EMFs), used as a physiotherapy for bone defects, have been suspected to cause potential hazards to human health due to the long-term exposure. To optimize the application of EMF while avoiding its adverse effects, a combination of EMF and tissue engineering techniques is critical. Furthermore, a deeper understanding of the mechanism of action of EMF will lead to better applications in the future.

**Methods:**

In this research, bone marrow mesenchymal stem cells (BMSCs) seeded on 3D-printed scaffolds were treated with sinusoidal EMFs in vitro. Then, 5.5 mm critical-sized calvarial defects were created in rats, and the cell scaffolds were implanted into the defects. In addition, the molecular and cellular mechanisms by which EMFs regulate BMSCs were explored with various approaches to gain deeper insight into the effects of EMFs.

**Results:**

The cell scaffolds treated with EMF successfully accelerated the repair of critical-sized calvarial defects. Further studies revealed that EMF could not directly induce the differentiation of BMSCs but improved the sensitivity of BMSCs to BMP signals by upregulating the quantity of specific BMP (bone morphogenetic protein) receptors. Once these receptors receive BMP signals from the surrounding milieu, a cascade of reactions is initiated to promote osteogenic differentiation via the BMP/Smad signalling pathway. Moreover, the cytokines secreted by BMSCs treated with EMF can better facilitate angiogenesis and osteoimmunomodulation which play fundamental roles in bone regeneration.

**Conclusion:**

In summary, EMF can promote the osteogenic potential of BMSCs and enhance the paracrine function of BMSCs to facilitate bone regeneration. These findings highlight the profound impact of EMF on tissue engineering and provide a new strategy for the clinical treatment of bone defects.

## Background

Bone, as one of the most extensive and universal organs in the human body, is of great significance to human health. The failure of bone function not only leads directly to a reduced quality of life but also imposes a staggering financial burden on the health care system [1–3]. Bone grafting is a common surgical method to augment bone regeneration [4]. Among all clinically available grafts, autologous bone is still considered the gold standard. Nevertheless, autologous bone grafts possess the disadvantages of poor availability, donor-site morbidity and prolonged operation time [5–7].

In an effort to circumvent these limitations, bone tissue engineering (BTE) has grown in popularity and is currently being studied as a possible alternative to fracture management [8]. With the development of BTE, an increasing number of new materials have been developed as bone substitutes. In addition, the selection of stem cell sources, growth factors and mechanical stimulation is continuously being optimized to boost the osteogenic properties of synthetic bone.

Bone marrow mesenchymal stem cells (BMSCs) are currently the most common source of stem cells in bone tissue engineering due to their relative ease of acquisition, high proliferative ability and established regenerative potential [9, 10]**.** Moreover, BMSCs have been extensively studied and found to enhance angiogenesis by secreting pro-angiogenic factors and play a vital role in osteoimmunomodulation [11–14].

The electrical environment is one of the most important microenvironments in which bone tissue exists. The outer electromagnetic fields (EMFs) have a vital effect on bone tissue and osteoblasts. Since Bassett et al. first proposed in 1974 that electromagnetic fields can promote osteogenesis [15], the effect of EMF on bone regeneration has been extensively studied. EMF has been successfully employed as an adjunctive therapy for the treatment of orthopedic disorders such as spinal cord injury, fresh fractures and delayed fractures [16–18]. However, prolonged exposure to EMF can lead to an increased risk of certain cancers, Alzheimer’s disease, male infertility and other conditions [19]. This may be attributed to the genotoxic effects, neurological effects and carcinogenicity of EMF [20–22]. The combination of EMF and tissue engineering technology may optimize clinical treatments with EMF in bone regeneration while avoiding its adverse effects. More importantly, deep insights into the mechanisms by which EMF regulate BMSCs will improve the use of EMF in the future. Therefore, in this research, a 3D-printed scaffold loaded with BMSCs stimulated by low-frequency sinusoidal electromagnetic fields (15 Hz, 0.3 mT) was utilized to repair bone defects, and its role in bone repair was examined in detail. The selection of EMF parameters was based on the previous research by our team [23–25].

It has been widely reported that a PCL (polycaprolactone)/HA (hydroxyapatite) composite material demonstrates enhanced osteogenic ability in bone repair [26, 27] and that polydopamine coating can improve cell adhesion [28, 29]. Therefore, in this study, polydopamine-coated scaffolds made of PCL and HA were used as cell carriers. Moreover, the molecular and cellular mechanisms by which sinusoidal EMF regulates BMSCs were investigated in vitro. To date, all research that has been conducted has aimed at exploring the regulatory effects of EMF on BMSCs and optimizing the application of EMF in the clinic while avoiding its adverse effects.

## Materials and methods

### Manufacture of PCL/HA hybrid scaffolds

PCL and HA were dissolved in an excess of dichloromethane at a ratio of 7:3 and the solution was mixed uniformly on a magnetic stirrer. Over several hours of volatilization of methylene chloride at room temperature, the solution gradually became viscous. Then, a cross-linked porous scaffold was fabricated layer by layer with an FDM (fused deposition modeling) 3D printer. The size of the scaffold used in vitro was 8 mm in length and 1 mm in thickness. These porous scaffolds were cut into discs with a diameter of 5.5 mm for in vivo trials. Finally, the scaffolds were placed in a draught cupboard overnight to vapourize the remaining methylene chloride.

### Polydopamine surface coating

The method for polydopamine coating has previously been reported in “Science” [30]. In brief, dopamine powders were dissolved in 10 mM Tris-HCl (pH 8.5) to prepare a 2 mg/ml dopamine Tris-HCL solution. Immediately afterwards, the porous scaffolds were immersed into the dopamine Tris-HCL solution and stirred overnight at room temperature. The scaffold surface modified by polydopamine changed from white to brown. The coated scaffolds were rinsed with ultrapure water 3 times and placed in a drying oven. Finally, these scaffolds were sent to the hospital for sterilization with ethylene oxide.

### Cell culture

Rat bone marrow mesenchymal stem cells *purchase*d from Cyagen Biotechnology Co., Ltd. (Suzhou, China) were cultured in α-MEM supplemented with 10% foetal bovine serum (Gibco, 10091148, NY, USA), 100 U/ml penicillin and 100 U/ml streptomycin (Sigma-Aldrich, A5955, USA). Then, 10 mM β-glycerophosphate, 50 mg/ml ascorbic acid and 10 nM dexamethasone were added to complete α-MEM to prepare osteogenic medium (OM). On a vertical flow clean bench, sterilized scaffolds were placed in 24-well plates and washed 3 times with phosphate-buffered saline (PBS). Next, they were incubated in α-MEM without foetal bovine serum for 2 h before cell seeding. BMSCs at passage 3 were seeded on the scaffolds and cultured with α-MEM complete medium at 37 °C, 5% CO^2^ and 95% humidity. Then, cell-seeded scaffolds were transferred to new 24-well plates the next day, and the culture medium was changed every 2 days.

### Sinusoidal electromagnetic field stimulation system

**Fig. 1:**
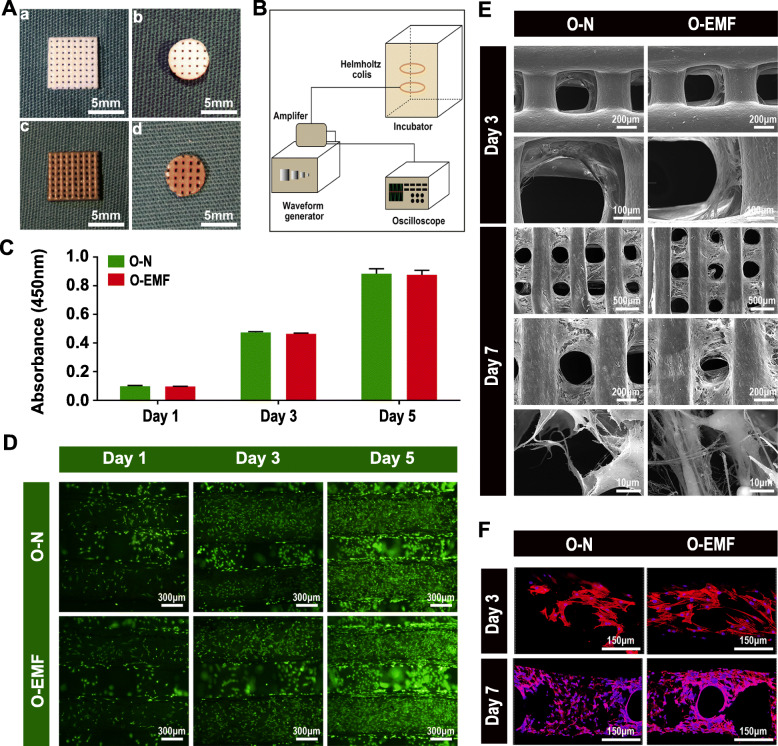
Detection of biocompatibility of scaffolds. **A** The appearance and structure of scaffolds. (a) (b) Scaffolds made of hydroxyapatite and polycaprolactone via 3D printing. (c) (d) PCL/HA scaffolds coated with polydopamine. **B** Sinusoidal electromagnetic fields generation system. **C** The proliferation of BMSCs after culturing for 1, 3 and 5 days on the scaffolds detected by the CCK-8 kit. **D** Live-dead staining images of BMSCs after culturing for 1, 3 and 5 days on the scaffolds. **E** Morphology of BMSCs seeded on the scaffold after culturing for 3 and 7 days observed by SEM. **F** Cytoskeleton of BMSCs seeded on the scaffold for 3 days and 7 days observed by confocal microscope

The EMF stimulation system that has been used in our previous studies [31, 32] consists of an EMF generator, an amplifier, an oscilloscope and a pair of Helmholtz coils that are 30 cm in diameter and 15 cm apart (Fig. 1b). The system was designed and manufactured by the Naval University of Engineering (Wuhan, China). EMFs with adjustable intensity and frequency (0–5 mT, 1–200 Hz) were generated by these devices. Helmholtz coils were positioned in an incubator with 5% CO_2_ and 95% humidity at 37 °C. Cell-seeded scaffolds assigned to the N-EMF and O-EMF groups were exposed to a sinusoidal electromagnetic field (15 Hz, 0.3 mT) for 4 h each day.

### Cell viability

BMSCs (4 × 10^4^) were seeded into a 24-well plate in which a scaffold was ensconced, and the proliferation of cells was detected by Cell Counting Kit-8 assay (CCK-8, Dojindo, CK04, Japan) after culturing for 1, 3 and 5 days. In brief, complete α-MEM and CCK-8 solution were mixed at a ratio of 9:1 to configure a working solution. Two milliliters working solution was added to each well to submerge the scaffold before the cell-seeded scaffolds were incubated at 37 °C for 2 h. The absorbance was measured at 450 nm using a microplate reader (Bio-Rad, USA). A live-dead kit (Thermo Fisher, L3224, USA) was used to evaluate cell cytotoxicity according to the instructions after 1, 3 and 5 days of culture. The BMSCs were imaged using a fluorescence microscope (EVOS FL Auto, Life Technologies, USA).

### Cell morphology

BMSCs (4 × 10^4^) were seeded into a 24-well plate in which a scaffold was ensconced. After 3 days and 7 days of culture under the appropriate conditions, the cell-seeded scaffolds were washed twice with PBS and fixed with 2.5% glutaraldehyde at 4 °C for 4 h. Then some of the cell scaffold constructs were dehydrated through an ethanol gradient (50, 60, 70, 80, 90 and 100%), followed by drying in vacuum. The dehydrated samples were coated with a layer of gold by an ion sputtering instrument (Q150R S, Quorum Technologies, UK) prior to scanning electron microscopy (SEM) observation (VEGA 3 LMU, Tescan, CZ); the remaining scaffolds were permeabilized with 0.5% Triton X-100 solution for 5 min and then washed 2 times with PBS. Subsequently, the samples were soaked with the prepared TRITC-phalloidin working solution (Solarbio, CA1610, Beijing, China) and incubated at room temperature for 30 min in the dark. After washing 3 times in PBS, the nuclei were stained with 100 nM DAPI (Solarbio, C0060, Beijing, China) solution for 30 s. Finally, the processed samples were observed with a confocal microscope (Eclipse, NIKON, Japan).

### Modelling of calvarial defects in rats

Fifteen male SD rats (10 weeks, 300–350 g) were provided by the Laboratory Animal Center of Tongji Hospital. All procedures were approved by the Animal Care and Use Committee of Huazhong University of Science and Technology. The provided rats were used to create a cranial bone defect model for an in vivo study as previously described [33]. In brief, the rats were anaesthetized with 5% chloral hydrate. After shaving and disinfecting the surgical site, the skin and subcutaneous tissue were cut to expose the skull. Then two penetrating skull defects with a diameter of 5.5 mm were created on both sides of the midline of the skull with a ring drill. The periosteum was completely removed from the skull surface by scraping, while the dura mater remained intact. Cell scaffolds that had been cultured for 1 week in OM with or without EMF were embedded in the defect site. The scaffolds were covered with soft tissue, and the incision was sutured with surgical sutures. The 15 operated rats were divided into 3 groups (*n* = 10) and treated as follows: (1) Blank group: Nothing was implanted into the defective skull. (2) O-N group: The cell scaffolds cultured with OM were implanted in the defect sites. (3) O-EMF group: Rats were implanted with cell scaffolds treated by EMF under OM. All of the rats were euthanized by rapid injection of an excess of 10% chloral hydrates at 8 weeks after surgery. Retrieved samples were fixed in 10% formalin for 2 days and then transferred to 70% ethanol for further analysis.

### Computed tomography (CT) and histological analysis

A Bruker Micro-CT SkyScan 1276 system (Kontich, Belgium) with an isotropic voxel size of 6.5 μm was used to image the skull, and reconstruction was accomplished by NRecon (version 1.7.4.2). The percentage of new bone volume relative to tissue volume (BV/TV) and the bone mineral density (BMD) were calculated by CT Analyser software (version 1.18.8.0) to assess the healing of bone defects. After this assessment, specimens were processed into 50-μm-thick undecalcified sections and stained with haematoxylin-eosin (H&E), Masson trichrome and von Kossa to further explore the bone formation in the defect sites in each group. The new bone area fraction, calculated as the new bone area/defect area within the defect of each section, was obtained using ImageJ software (*n* = 10).

### Alkaline phosphatase (ALP), collagen and mineralization assays

To further explore the mechanism by which EMF regulates BMSCs, the cells were randomly distributed into four groups: (1) N-N group: cells were cultured in normal medium (α-MEM) without EMF intervention; (2) N-EMF group: cells were cultured in normal medium with EMF intervention; (3) O-N group: cells were cultured in osteogenic medium without EMF intervention; (4) O-EMF group: cells were cultured in osteogenic medium with EMF intervention. A total of 1 × 10^4^ cells were seeded in 24-well plates and fixed with 4% paraformaldehyde after 7 days of culture. ALP and collagen were stained with a BCIP/NBT alkaline phosphatase colour development kit (Beyotime, China) and Sirius red (Sigma, USA), respectively. Mineralization was detected by Alizarin Red (Cyagen, China). The staining-positive area fraction, calculated as the staining-positive area/total area, was obtained using ImageJ software (*n* = 6).

### Analysis of gene expression

A total of 2 × 10^5^ cells were seeded into a 24-well plate positioned with a scaffold, and intervention was started the day after the scaffold was transferred to a 12-well plate. Total RNA was extracted from the cell scaffold constructs using the RNeasy Kit (Omega, R6834-01, USA) after 4 days of culture. A total of 1 μg of RNA was reverse transcribed using the Reverse Transcriptase Kit (Toyobo, FSQ-101, Japan). Relative expression of bone morphogenetic protein 2 (BMP2), bone sialoprotein (BSP), runt-related gene 2 (Runx2), osteocalcin (OCN) and osteopontin (OPN) was detected by quantitative real-time PCR (RT-qPCR). Gene-specific primers (Tables 1 and 2) purchased from Tsingke Biotechnology Company (Beijing, China) were used to amplify the cDNA in a Bio-Rad myiQ2 thermal cycler (Bio-Rad, Hercules, CA, USA), of which GAPDH was used as the internal control for target mRNA. The RT-qPCR cycling conditions were 95 °C for 30 s followed by 40 cycles of 94 °C for 5 s and 60 °C for 35 s. Melt curve analyses were performed on each primer set to minimize primer-dimers and nonspecific products. The 2^**–△△Ct**^ method was used to analyse the relative expression of target mRNA. The experimental grouping is the same as in section “Alkaline phosphatase (ALP), collagen and mineralization assays”.

**Table 1:** Specific primers used in this study

Gene (rat)		Primer sequences
Smad1	Forward: Reverse:	CAGCCCTTTTCAGATGCCAG ACTGCTTGAACATCTCCTCTATTG
Smad4	Forward: Reverse:	GGTCCGTAGGTGGAATAGCC AATCCAGCACGGGGTTTCTT
Smad5	Forward: Reverse:	CTGCCAATAACAAGAGCCGC ACCTCCCCACCAACGTAGTA
Smad8	Forward: Reverse:	AACAACCAGCTCTTCGCCC CTGGCGATGATACTCGGCTC
ACVR1	Forward: Reverse:	GACTGTACGCTGTCAGGCTC CCATACTCGGGGAAGGGAGA
BMPR1A	Forward: Reverse:	GCACCAGAGGACACCTTACC GCTGGGCTTTTGGTGAATCC
BMPR1B	Forward: Reverse:	TTCTTCACCACGGAGGAAGC AGTCCAAGACCCAGTCCCTT
BMPR2	Forward: Reverse:	GAAGAGCACAGAGGCCCAAT CCTGATTTGCCATCCTGCGT
BMP2	Forward: Reverse:	GAAGAAATTGCAAAATGAAGACTGC CGCCATCTCCATTTTCTTCCG
BSP	Forward: Reverse:	GAAGAAATTGCAAAATGAAGACTGC CGCCATCTCCATTTTCTTCCG
Runx2	Forward: Reverse:	CTACTCTGCCGAGCTACGAAAT TCTGTCTGTGCCTTCTTGGTTC
OPN	Forward: Reverse:	CCAGCCAAGGACCAACTACA CCAAGTGGCTACAGCATCTGA
OCN	Forward: Reverse:	GGAGGGCAGTAAGGTGGTGA GAAGCCAATGTGGTCCGC
VEGFA	Forward: Reverse:	GGCCATCAAGCTCTCTCCTC CACACACAGCCAAGTCTCCT
FGF2	Forward: Reverse:	TGTCCATCAAGGGAGTGTGTG TCGTTTCAGTGCCACATACCA
VWF	Forward: Reverse:	GGTGGAGGAAGACCCCATTG GATGTCCAGGTATGGCTCGG
eNOS	Forward: Reverse:	GAAGGTCGGTGTGAACGGAT CCCATTTGATGTTAGCGGGAT
GAPDH	Forward: Reverse:	GAAGGTCGGTGTGAACGGAT CCCATTTGATGTTAGCGGGAT

**Table 2:** Specific primers used in this study

Gene (mouse)		Primer sequences
IL-1	Forward: Reverse:	CCTGGACTGTGAGCATGGATA GTAAGGGGCGTCATCAGGAC
IL-6	Forward: Reverse:	GGTGCCCTGCCAGTATTCTC GGCTCCCAACACAGGATGA
CD206	Forward: Reverse:	CTCTGCCATCACGTTTAGTGAA GACGGTTATCAAAACAACGCC
PDGFB	Forward: Reverse:	CATCCGCTCCTTTGATGATCTT GTGCTCGGGTCATGTTCAAGT
VEGFA	Forward: Reverse:	CCACCTGCAAGACCATCGAC CTGGCGAGCCTTAGTTTGGAC
TGF-β	Forward: Reverse:	TATTCAGCGGACTCACCAGC AACCAACCTCCTCAAACCGT
GAPDH	Forward: Reverse:	TTCCAGGAGCGAGACCCCACTA GGGCGGAGATGATGACCCTTTT

### Western blot analysis

The proteins were analysed from whole-cell lysates of cells cultured in each group after 7 days. The protein concentration was measured by BCA protein assay reagent (Boster, Wuhan, China). Then 40 μg protein samples were separated by SDS-polyacrylamide gels and transferred to PVDF membranes. The membranes were then blocked with 5% bone serum albumin for 1 h and incubated with primary antibodies (COL1, BMP2 at 1:1000 dilution, β-actin at 1:5000 dilution, Abcam, UK; OCN at 1:1000 dilution, Santa Cruz Biotechnology) at 4 °C overnight. Next, blots were incubated with horseradish peroxidase (HRP)-conjugated secondary antibodies (goat anti-rabbit and goat anti-mouse antibodies at a 1:5000 dilution, Boster, China) for 1 h. The bands were detected by the Western ECL Substrate Kit (Thermo Pierce, USA). The proteins were normalized to β-actin. The experimental grouping is the same as in section “Alkaline phosphatase (ALP), collagen and mineralization assays”.

### Detection of BMP signalling pathway

The BMP signalling pathway is well known as an important pathway that can regulate cell fate in developing and mature tissues [34–36]. Moreover, BMP signalling is necessary and sufficient for osteogenesis [37–39]. To understand whether EMF affects the osteogenic properties of BMSCs by regulating the BMP signalling pathway, RNA of the cells stimulated by EMF for 4 h was immediately extracted. Subsequently, the expression levels of genes related to the BMP signalling pathway [type IA BMP receptor (BMPR1A/ALK3), type IB BMP receptor (BMPR1B), type II BMP receptor (BMPR2), type I activin receptor (ACVR1/ALK2), Smad1/5/8, Smad4)] were assayed by RT-qPCR. Furthermore, LDN193189, a selective BMP type I receptor inhibitor, was added to the N-EMF and O-EMF groups at a concentration of 100 nM before the cells were stimulated by EMF to verify the regulatory effect of EMF on the BMP signalling pathway. In addition to the four groups in section “Alkaline phosphatase (ALP), collagen and mineralization assays”, the two newly established groups were represented by N-EMF-I and O-EMF-I.

### Immunofluorescence staining

BMSCs grown on coverslips were fixed in 4% paraformaldehyde and permeabilized with 0.5% Triton X-100 for 15 min. Then the coverslips were blocked with ready-to-use goat serum for 30 min. Immunostaining was carried out using primary antibodies including rabbit anti-phospho-Smad1/5/8 (1:100, Cell Signaling Technology), mouse anti-BMPRIB (1:50, Santa Cruz Biotechnology), rabbit anti-BMPRII (1:100, Absin Biosciences Inc., China), rabbit anti-Runx2 (1:100, Cell Signaling Technology), mouse anti-OPN (1:50, Santa Cruz Biotechnology) and mouse anti-OCN (1:50, Santa Cruz Biotechnology). The secondary antibodies were CY3-conjugated goat anti-rabbit IgG (1:200, Boster, China) and FITC-labeled goat anti-mouse IgG (1:200, Boster, China). The staining results were imaged on a confocal microscope (Eclipse, NIKON, Japan). Cell fluorescence quantitative analysis was performed by ImageJ software (*n* = 6).

### Collection of conditioned medium

To explore the effect of EMF on the paracrine function of BMSCs, the collection of conditioned medium from BMSCs was necessary. After the cell scaffolds of the O-N and O-EMF groups were cultured for 4 days, the medium was discarded and replaced with α-MEM and incubated for another 4 days in a normal environment. Subsequently, the medium was collected, and 10% FBS was added to generate a conditioned medium for the culture of the murine-derived macrophage cell line RAW264.7 (RAW) and endothelial progenitor cells (EPCs). Both cell lines were purchased from Zhong Qiao Xin Zhou Biotechnology Co., Ltd. (Shanghai, China).

### Angiogenic effects of BMSCs

After culturing EPCs with conditioned medium for 2 days, RT-qPCR was used to measure the expression level of angiogenic genes [von Willebrand factor (vWF), endothelial nitric oxide synthase (eNOS), fibroblast growth factors (FGF), vascular endothelial growth factor-A (VEGFA)]. At the same time, cells cultured with α-MEM were used as a control group. To intuitively understand the effect of BMSCs on angiogenesis, we cultured EPCs grown on Matrigel with conditioned medium and observed tube formation at different times. Generally, the 100 μl of Matrigel (*Corning, USA*) was placed into a 48-well plate, then 2 × 10^4^ cells were added to each well including 1 ml of conditioned medium with α-MEM as the control. Angiogenesis status was imaged under a microscope (Leica) after 4 h and 6 h of incubation. After 6 h of culture, the cells were stained with a live-dead kit (L3224, Thermo Fisher) and observed under a fluorescence microscope (EVOS FL Auto, Life Technologies, USA). The total length, number of segments and nodes in 6 randomly chosen fields were quantified using the Angiogenesis Analyzer macro in ImageJ [40].

### Osteoimmunomodulation of BMSCs

To simulate the inflammatory environment in the early stage of fracture, lipopolysaccharide (LPS, 1 μg/ml, Sigma) was added to the culture medium to activate RAW cells for 3 h. Next, conditioned medium from the O-N and O-EMF groups was used to culture RAW cells. The cells cultured in α-MEM were defined as the control group, and the inactive RAW cells were used as the blank group to be cultured in α-MEM. After 1 day of incubation, the expression levels of macrophage polarization-related genes [interleukin-6 (IL-6), interleukin-1β (IL-1), CD206, transforming growth factor-β1 (TGF-β), platelet-derived growth factor-B (PDGFB), VEGFA)] were detected by RT-qPCR.

Cell surface markers (CD86 and CD206) related to macrophage polarization were detected by flow cytometry. In detail, each group of cells treated with the above methods was collected. Then RAW cells were washed 3 times with PBS and resuspended in 1% bovine serum albumin (BSA) for 15 min at 4 °C to block nonspecific antigens. Next, APC-CD206 antibody (0.25 μg/test, Thermo Fisher Scientific) and PE-CD86 antibody (0.125 μg/test, Thermo Fisher Scientific) were incubated with the RAW cells for 15 min at ambient temperature. After washing twice with PBS, cells were resuspended in 100 μl of 1% BSA and analysed on a Guava flow cytometer (Millipore, USA). Data were analysed by Flowjo**_**V10 software.

### Statistical analysis and image editing

To determine whether the differences between the two sets of data were statistically significant, a two-tailed homoscedastic *t*-test was applied. *^, #, ^, &^*P* < 0.05 was considered to be statistically significant; and **^, ##, ^^, &&^*P* < 0.01 was considered to be extremely significant; otherwise, results were considered not significant. Values are reported as the mean ± standard deviation (SD). All in vitro experiments were performed at least three times. Brightness and contrast were adjusted equally across all images for improved visibility.

## Results

### Characterization of the porous scaffold

As shown in Fig. 1Aa, a porous scaffold with a side length of 8 mm and a height of 1 mm was manufactured by a 3D printer. The principal components of the scaffold are PCL and HA because PCL is a promising material for bone formation, and the presence of HA improves osteoblast activity [41]. Then, we processed the scaffold into a disc with a diameter of 5.5 mm and a thickness of 1 mm to meet the requirements for animal experiments (Fig. 1Ab). To increase the cell adhesion property of the scaffolds, the surface was coated with a layer of polydopamine [42]. The polydopamine particles were evenly distributed and bound to the surface of the scaffolds (Fig. 1Ac, d).

### Cells viability and morphology on scaffolds

The proliferation of BMSCs seeded on scaffolds from the 4 groups was detected by CCK-8 after culturing for 1, 3 and 5 days. As shown in Fig. 1C, cells proliferated over time, but there was no significant difference between each group. Similar to the cell proliferation test, the live-dead test (Fig. 1D) also showed that cells seeded on scaffolds were healthy regardless of the EMF stimulation.

In addition to cell viability, the morphology of cells that adhered to the scaffolds was observed with SEM (Fig. 1E) and confocal microscopy (Fig. 1F). Most cells were completely attached to the surface of the scaffold on the 3rd day, while a small number of cells hung in the corner of the frameworks. By the 7th day, the scaffolds were densely covered with the cells. This illustrated that polydopamine-coated PCL/HA scaffolds exhibit superior biocompatibility, while EMF had little consequence on the proliferation and activity of BMSCs.

### Bone regeneration in vivo

**Fig. 2:**
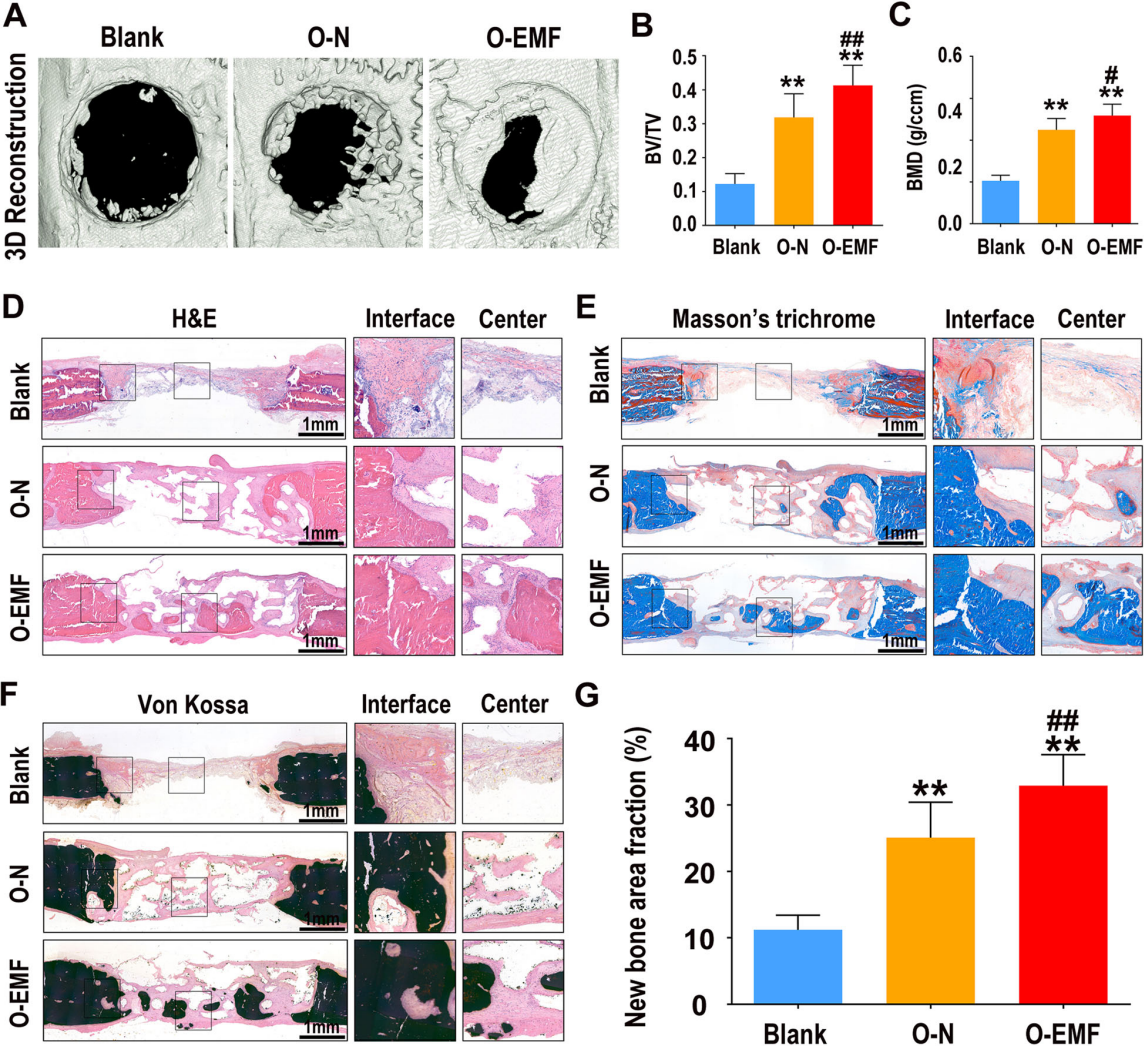
Evaluation of bone regeneration in vivo. **a** 3D reconstruction images of the cranial bone defects at 8 weeks. **b**, **c** Ratio of bone volume to total volume (BV/TV) and bone mineral density (BMD). **d, e, f** Tissue sections stained with H&E, Masson trichrome and Von kossa. **g** Quantification analysis of new bone area fraction in different groups at 8 weeks (*n* = 10). *P < 0.05 compared to Blank, **P < 0.01 compared to Blank, ^#^*P* < 0.05 compared to O-N, ^##^*P* < 0.01 compared to O-N

Animal trials were conducted to verify whether the combination of EMF and tissue engineering techniques can better promote bone regeneration in vivo. The 3D reconstructed rat craniums at 8 weeks are shown in Fig. 2a. Accordingly, there was almost no new bone formation in the blank group. Quite a few new bones growing from defect margins were observed in the O-N group, with limited newly formed bone in the centre. In the O-EMF group, extensive new bone formation was observed at the centre and periphery of the defect, indicating osseointegration of the new bone with the defect margins. The statistical results of BV/TV (Fig. 2b) and BMD (Fig. 2c) also support this argument. The sections stained with H&E (Fig. 2d), Masson’s trichrome (Fig. 2e), von Kossa (Fig. 2f) and the statistical analysis (Fig. 2g) revealed the same repair effects as the above tests. All experimental results indicated that 3D-printed scaffolds loaded with BMSCs treated by EMF accelerated bone regeneration.

### Osteogenic differentiation of BMSCs on scaffolds

**Fig. 3:**
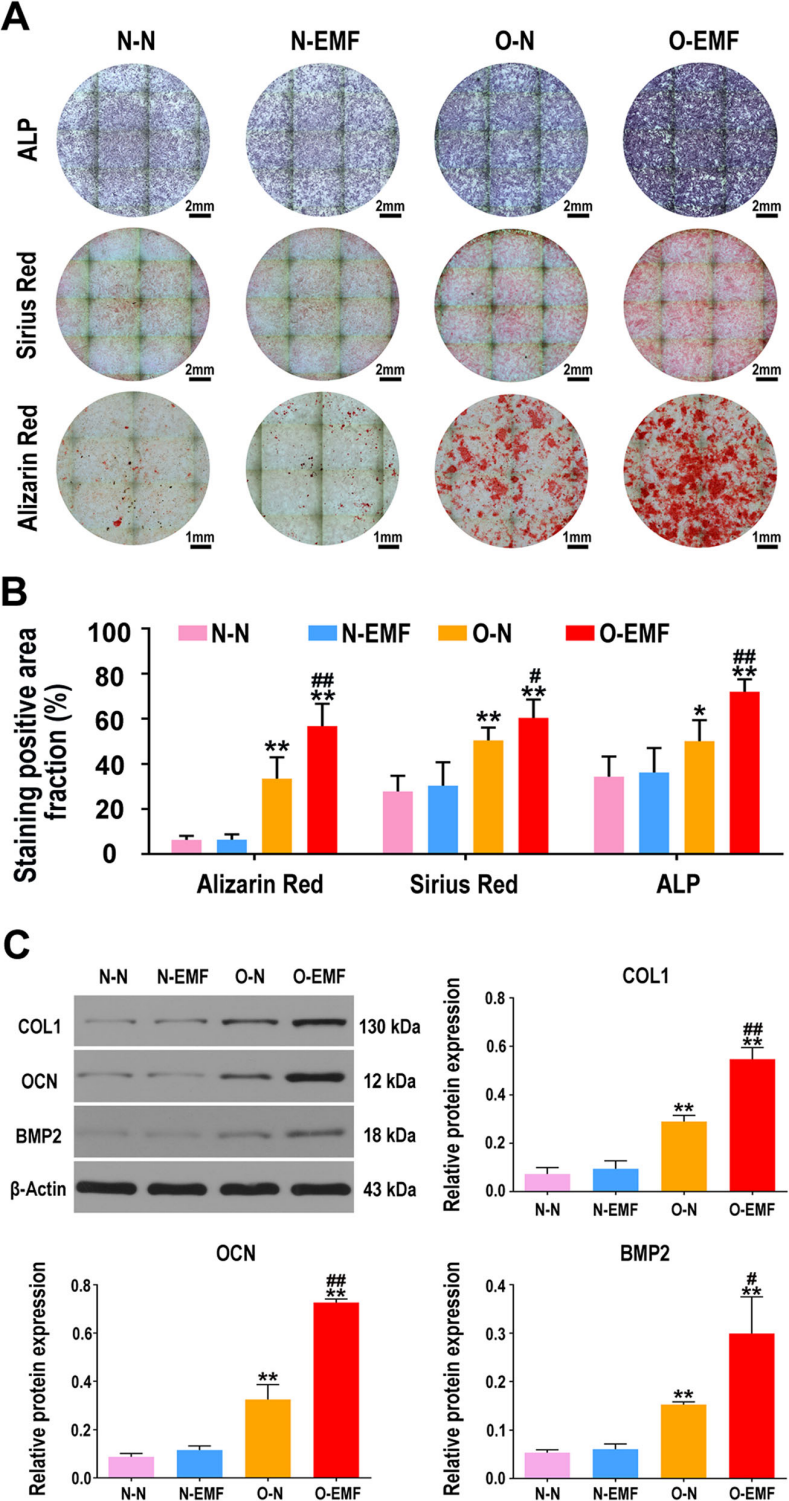
Regulation of EMF on osteogenic differentiation of BMSCs. **a** Images of ALP, Sirius Red and Alizarin Red staining of BMSCs seeded in 24-well plates after culturing for 1 week. **b** Semi-quantitative analysis of ALP, Sirius Red and Alizarin Red staining among both groups (*n* = 6). Data shown as mean ± SD. **c** Effects of EMF on osteogenic protein expression of BMSCs seeded on scaffolds after culturing for 1 weeks (COL1, OCN and BMP2) detected by western blotting. *P < 0.05 compared to N-N, **P < 0.01 compared to N-N, ^#^*P* < 0.05 compared to O-N, ^##^*P* < 0.01 compared to O-N

**Fig. 4:**
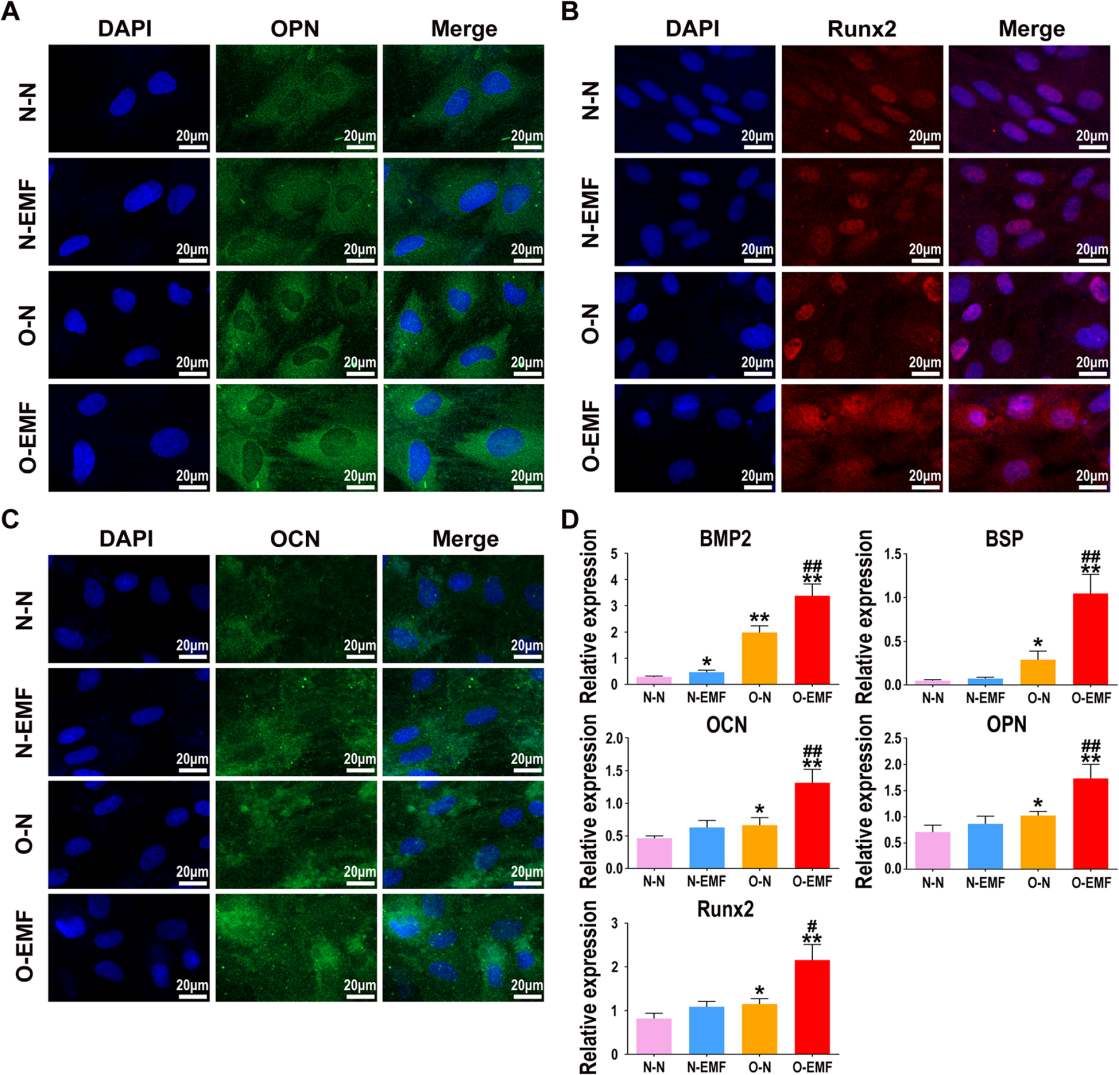
Regulation of EMF on osteogenic differentiation of BMSCs. **a** Immunofluorescence labeling for OPN of BMSCs seeded on coverslips. **b** Immunofluorescence labeling for Runx2 in BMSCs seeded on coverslips. **c** Immunofluorescence labeling for OCN in BMSCs seeded on coverslips. **d** Effects of EMF on osteogenic gene expression of BMSCs seeded on scaffolds after culturing for 4 days (BMP2, BSP, OPN, OCN and Runx2) detected by RT-qPCR. *P < 0.05 compared to N-N, **P < 0.01 compared to N-N, ^#^*P* < 0.05 compared to O-N, ^##^*P* < 0.01 compared to O-N

Osteogenesis is the crux of bone regeneration. Numerous tests have been conducted to explore the osteogenic differentiation of BMSCs stimulated by EMF. ALP is used as an early indicator of osteogenesis. Therefore, the cells cultured for 7 days were stained with ALP staining reagent and Direct Red 80. As shown in Fig. 3a, b, the cells from the O-EMF group were labeled with *dark1er colours*, which represent higher expression levels of osteogenic indicators. When BMSCs were cultured in α-MEM, EMF (15 Hz, 0.3 mT) slightly increased ALP activity, collagen deposition and mineralization. These trends became more significant when BMSCs were cultured in OM. Consistent with the results of the above experiments, the expression of osteogenic genes (Fig. 4a) including bone morphogenetic protein 2 (BMP2), bone sialoprotein (BSP), runt-related gene 2 (Runx2), osteocalcin (OCN) and osteopontin (OPN), also confirmed that BMSCs treated with EMF showed more significant osteogenic capacity under OM. Moreover, to further demonstrate the regulatory effect of EMF on BMSCs, western blotting was performed after 1 week of incubation to measure the expression levels of osteogenic proteins (Fig. 3c). All indicators, comprising BMP2, COL1 and OCN, revealed that EMF did not directly induce osteogenic differentiation of BMSCs but promoted the osteogenic potential of cells, which would be fully realized under the induction of OM. The results of OPN, Runx2 and OCN immunofluorescence (Fig. 4a–c; Figure S1) also support this argument.

### Regulatory effects of EMF on the BMP signalling pathway

**Fig. 5:**
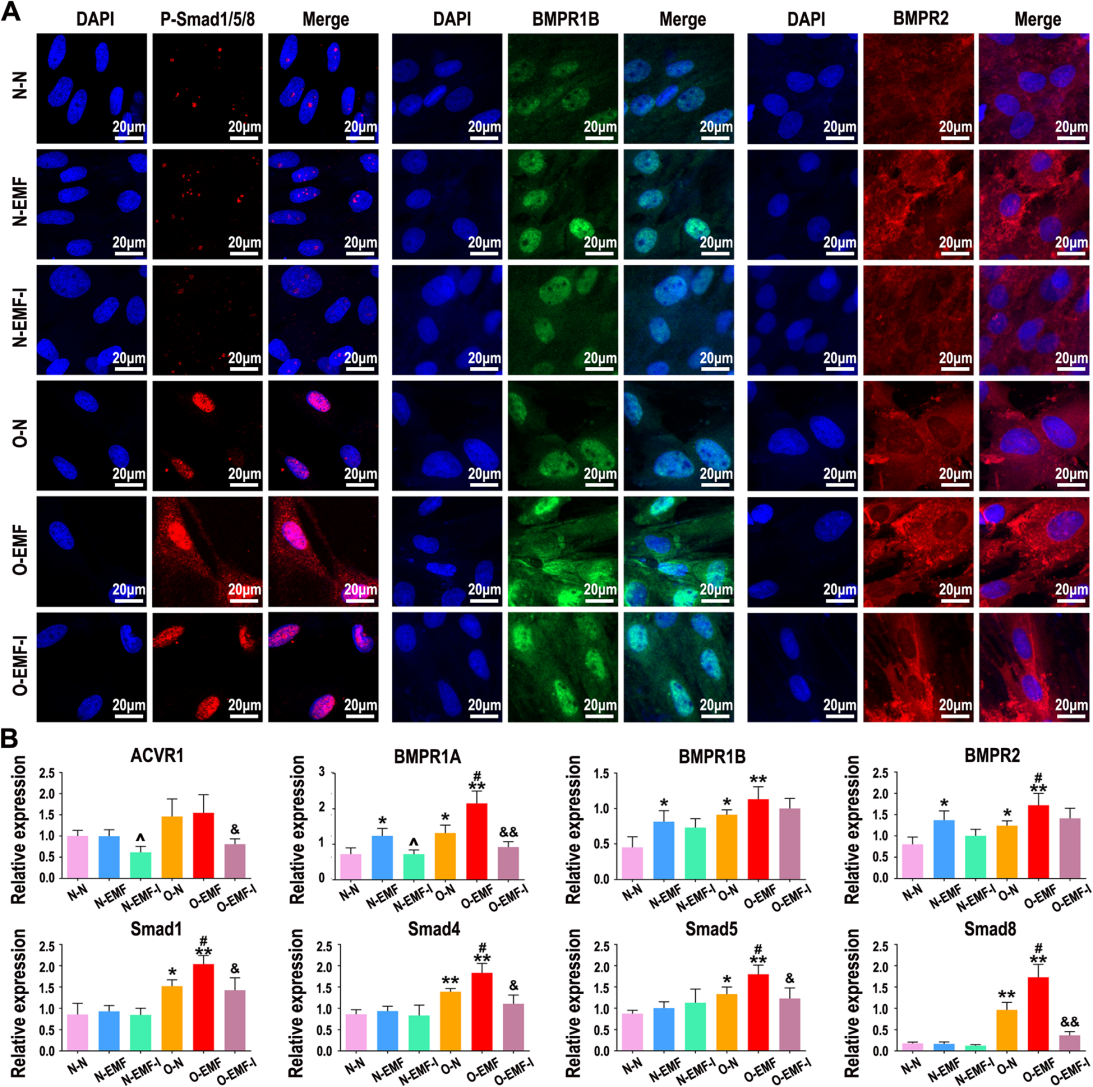
Regulation of EMF on BMP/Smad pathway. **a** Immunofluorescence labeling for P-Smad1/5/8, BMPR1B and BMPR2 in BMSCs seeded on coverslips. **b** Expression of ACVR1, BMPR1A, BMPR1B, BMPR2, Smad4 and Smad1/5/8 of BMSCs seeded on scaffolds were detected by RT-qPCR (*n* = 3). *P < 0.05 compared to N-N, **P < 0.01 compared to N-N, ^#^*P* < 0.05 compared to O-N, ^##^*P* < 0.01 compared to O-N, ^*P* < 0.05 compared to N-EMF, ^^*P* < 0.01 compared to N-EMF, ^&^*P* < 0.05 compared to O-EMF, ^&&^ *P* < 0.01 compared to O-EMF

The key proteins in the BMP signalling pathway were investigated to further explain the regulation of BMSCs by EMF. According to the experimental results (Fig. 5b), EMF significantly upregulated the gene expression of BMP receptors, including BMPR1A, BMPR1B and BMPR2, while ACVR1 did not seem to be affected by EMF. For Smad4 and Smad1/5/8, which are downstream of these receptors, upregulated genes were detected when BMSCs treated with EMF were cultured in OM. As expected, the effect of EMF on the Smad-dependent BMP pathway was significantly weakened with the participation of LDN193189. In addition, the immunofluorescence results of P-Smad1/5/8, BMPR1B and BMPR2 were similar to those of the PCR assay (Fig. 5a; Figure S2).

### Pro-angiogenic capacity of BMSCs treated by EMF

**Fig. 6:**
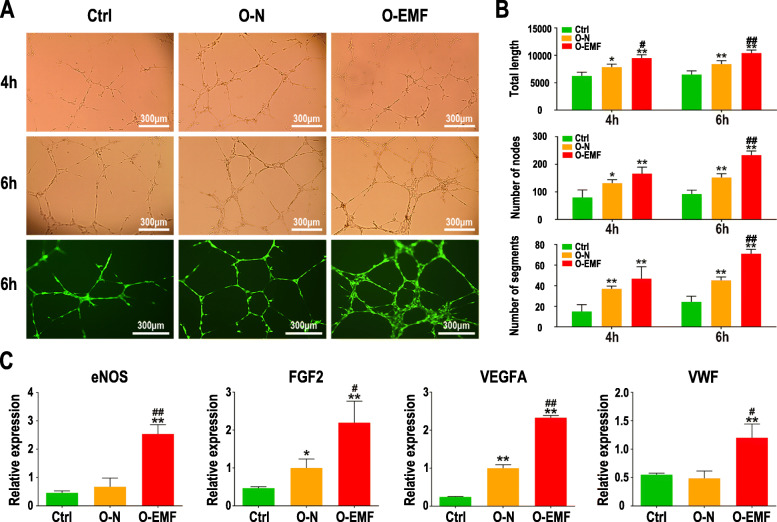
Effects of the conditioned medium of BMSCs on angiogenesis of EPCs. **a** Tube formation of EPCs grown on Matrigel exposed to α-MEM medium or conditioned mediums from O-N and O-EMF for 4 and 6 h. **b** Quantification of total length, nodes and segments of capillary network formed by EPCs after incubated for 4 and 6 h on the Matrigel (*N* = 6). **c** Effects of the conditioned medium of BMSCs on the angiogenic gene expression of EPCs after culturing for 2 days (eNOS, FGF2, VEGFA and VWF) detected by RT-qPCR (*n* = 3). *P < 0.05 compared to Ctrl, **P < 0.01 compared to Ctrl, ^#^*P* < 0.05 compared to O-N, ^##^*P* < 0.01 compared to O-N

According to the detection of angiogenic genes of EPCs assigned to three groups, it was clear that conditioned media promoted the angiogenesis of EPCs, and the angiogenic genes in the O-EMF group were upregulated almost twofold compared with those in the O-N group (Fig. 6c). At the same time, the conditioned media accelerated the formation of capillary-like structures in Matrigel during the 6 h of incubation. Both intuitive evaluation (Fig. 6a) and statistical data (Fig. 6b) suggested that conditioned medium of the O-EMF group exerted a more prominent effect in terms of promoting angiogenesis.

### Osteoimmunomodulation of BMSCs treated by EMF

**Fig. 7:**
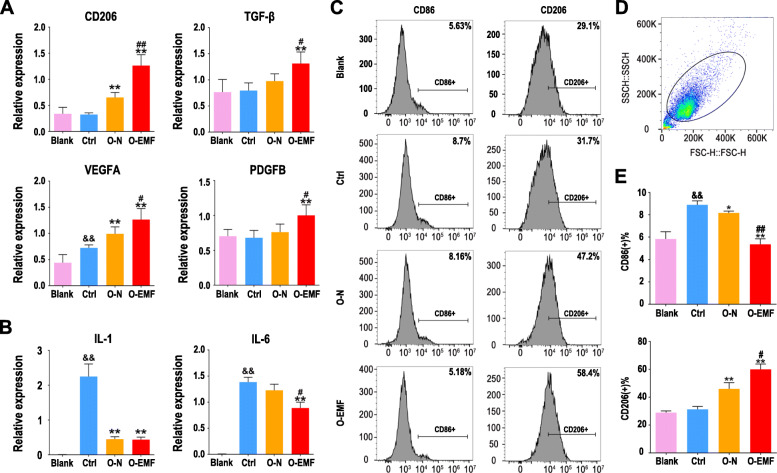
Effects of the conditioned medium of BMSCs on immunomodulation. **a** Real-time qPCR analyses of CD206, TGF-β, VEGFA and PDGFB mRNA expression in RAWs at day 1 (*n* = 3). **b** Real-time qPCR analyses of IL-1 and IL-6 mRNA expression in RAWs at day 1 (*n* = 3). **c** Results of flow cytometry, percentage of CD86-positive or CD206-positive cells. **d** Alive RAWs were gated based on forward scatter and side scatter. **e** Statistical results of CD86-positive and CD206-positive RAWs (*n* = 6). ^&^*P* < 0.05 compared to Blank, ^&&^*P* < 0.01 compared to Blank, *P < 0.05 compared to C, **P < 0.01 compared to C, ^#^*P* < 0.05 compared to O-N, ^##^*P* < 0.01 compared to O-N

Macrophages have an irreplaceable role in osteoimmunomodulation, which is a pivotal process in bone regeneration. After implantation, macrophages swiftly migrate to the interface between the graft and bone and manipulate the surrounding immune microenvironment in response to changes in the living environment [43]. The gene expressions of RAW cells cultured in conditioned media was evaluated by RT-qPCR. Compared to the blank group, the expression of genes associated with the proinflammatory M1 phenotype, such as IL-1 and IL-6, was significantly increased in the control group (Fig. 7b). This result indicated that we successfully simulated the early inflammatory environment at the fracture site. In the meantime, conditioned media downregulated the expression of proinflammatory genes, and the conditioned medium of the O-EMF group seemed to be more effective in terms of inhibiting inflammation. In addition, the expression of genes related to the pro-healing M2 phenotype in the O-EMF group was significantly higher than that in the control and O-N groups (Fig. 7a). The results of flow cytometry (Fig. 7c–e) were almost the same as those of RT-qPCR. RAW cells cultured with the conditioned media showed a clear tendency to differentiate to the M2 phenotype, and the conditioned medium of the O-EMF group was more effective at promoting the M2 phenotype over the M1 phenotype.

## Discussion

Seeded cells, cytokines and cellular biological scaffolds are crucial elements of bone tissue engineering [44, 45]. In recent decades, much attention has been given to BMSCs and their use for bone tissue engineering. Therefore, we believe that improving the multifunctionality of BMSCs is significant for accelerating the repair of bone defects. Our previous work demonstrated that sinusoidal EMF is a convenient and economical method to promote osteogenesis in BMSCs [24, 46]. Therefore, after many attempts, electromagnetic fields with specific parameters (15 Hz, 0.3 mT) were selected to modulate the pluripotency of BMSCs. At the same time, PCL/HA scaffolds coated with polydopamine were manufactured as cell vehicles.

In in vitro experiments, the expression of osteogenic genes (BMP2, BSP, OCN, OPN, Runx2) and osteogenic proteins (COL1, OCN, BMP2) and the staining of osteogenic markers (ALP, collagen, mineralization) indicated that EMF significantly improved the osteogenic ability of BMSCs cultured in OM. Nevertheless, it is worth considering that when BMSCs were cultured in normal medium, the effect of EMF on cells seemed to disappear. To clearly explain this strange phenomenon, we need to understand the mechanism by which EMF affects BMSCs.

TGF-β and BMP signalling play a fundamental role in embryonic bone development and postpartum bone homeostasis [47]. They regulate the differentiation of BMSCs by acting on the tetrameric receptor complex to trigger a cascade reaction downstream of the pathway. Many human bone diseases are attributed to the dysregulation of TGF-β and BMP signalling [48]. Therefore, we hypothesized that EMF affects the osteogenic differentiation of BMSCs by regulating the BMP signalling pathway. As a member of the TGF-β superfamily, BMP works by binding to type I and type II BMP receptors on the cell surface. After binding to the BMP ligand, the homodimer of the type II receptor and the homodimer of the type I receptor form a tetramer complex, which induces the transphosphorylation of the type I receptor [48, 49]. The phosphorylated type I receptor binds to Smad1/5/8 and enters the nucleus under the guidance of Smad4, where these proteins recruit cofactors and Runx2 to regulate the expression of osteogenic genes. In subsequent experiments, we found that EMF can upregulate the gene expression of BMPR1A, BMPR1B, BMPR2, Smad4 and Smad1/5/8 in BMSCs cultured with OM. The regulation of BMSCs by EMF was weakened with the participation of a BMP type I receptor inhibitor. These results confirmed our conjecture that EMF regulates the osteogenic differentiation of BMSCs through the BMP signalling pathway. However, in the absence of OM, EMF upregulated the expression of specific BMP receptors but did not enhance the expression of osteogenic indicators such as OPN, OCN and Runx2. This result suggested that the BMP/Smad signalling pathway was not fully activated. Therefore, we speculated that EMF could not induce the osteogenic differentiation of BMSCs directly but promoted their osteogenic potential by upregulating the quantity of specific BMP receptors. Although the increased receptor expression help cells receive extrinsic BMP signals, they cannot phosphorylate downstream components. Once these induced cells receive BMP signals from the surrounding milieu, the transmembrane receptors will bind to the signals to trigger cascade reactions through the BMP/Smad pathway. At this moment, the osteogenic potential of the stem cells is fully realized.

We also found that BMSCs stimulated by EMF contribute to the angiogenesis of EPCs. As a prosurvival factor of endothelial cells (ECs) both in vitro and in vivo, VEGF plays an indispensable role in the process of angiogenesis [50, 51]. VEGF binds to its corresponding receptor, VEGFR, and induces its homodimerization, which activates eNOS to realize the production of vascular nitric oxide (NO) [52]. The diffusion of NO in the vasculature not only contributes to vascular permeability but also promotes the proliferation and migration of ECs [53]. It has been reported that BMSCs can secrete angiogenic factors such as VEGF and erythropoietin [54]. This reasonably explains why the conditioned media of BMSCs enhanced the angiogenic ability of EPCs. Nevertheless, both *tube* formation assays and RT-qPCR experiments showed that the conditioned medium of BMSCs stimulated by EMF further increased the expression of angiogenic genes in EPCs. This may be attributed to EMF boosting the secretion of angiogenic factors by BMSCs. Thus, a scaffold loaded with BMSCs induced by EMF implanted into a bone defect may be more conducive to the angiogenesis of surrounding endothelial cells. FGF expressed in ECs acts together with Runx2 to promote the proliferation of osteoblasts and the execution of the osteogenic programme [55].

**Fig. 8:**
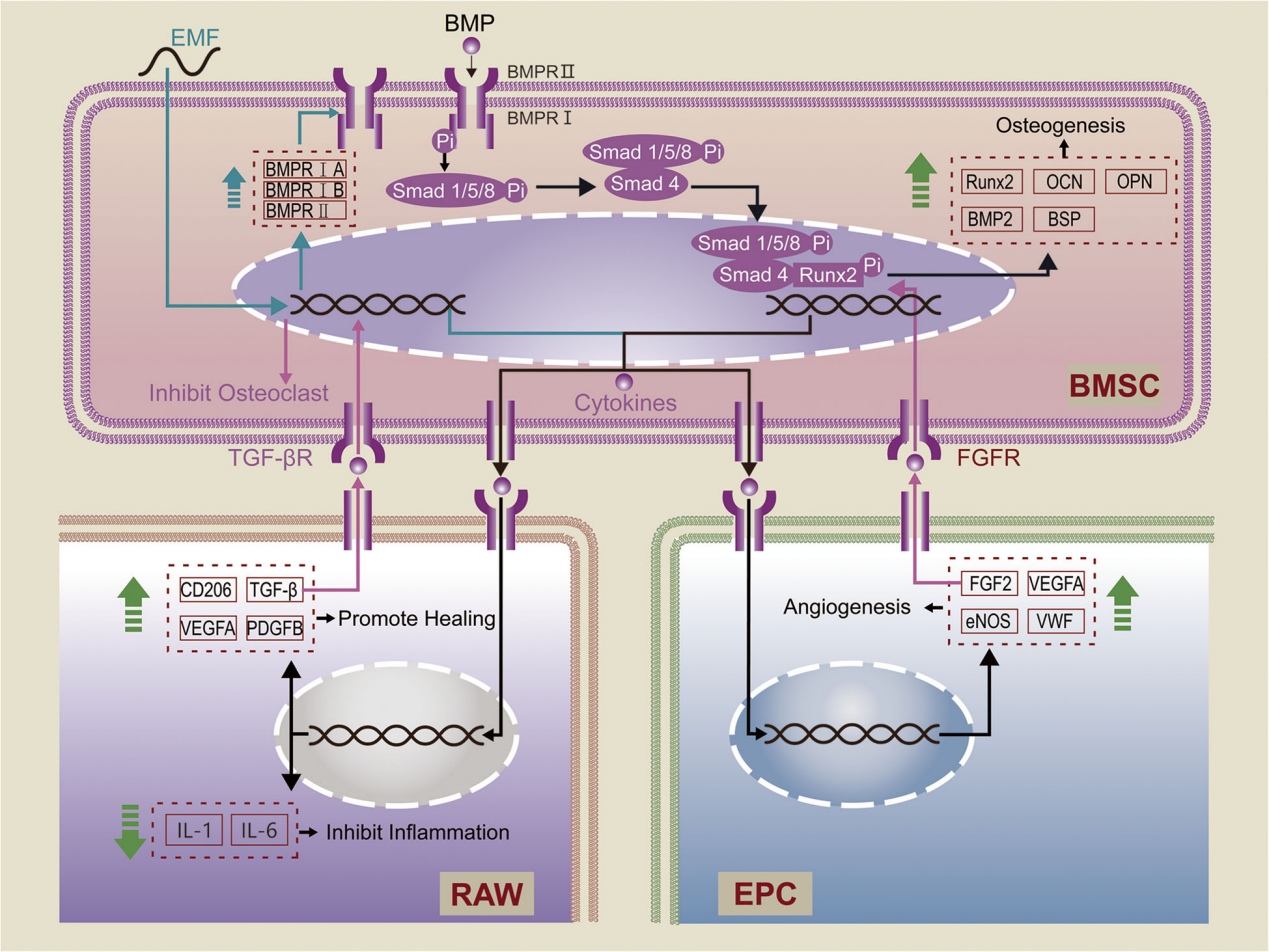
Explanation of the mechanism by which EMF motivates the multiple potential of BMSCs to facilitate osteogenesis, angiogenesis and osteoimmunomodulation

The effects of EMF on stem cells go far beyond the process described above. BMSCs stimulated by EMF positively regulate the immune microenvironment. Once bone defects occur, numerous macrophages gather around the wound and show considerable plasticity according to signals derived from the surrounding environment [43]. The M1 and M2 phenotypes represent two extremes of macrophage polarization [56]. In acute inflammation, M1 macrophages play a major role by promoting the migration of BMSCs and clearing cell debris and bacterial pathogens [57, 58]. However, the activated M1 phenotype stimulates inflammation by producing CD86 and iNOS (nitric oxide synthases), which are not conducive to bone regeneration. In contrast, the activated M2 phenotype inhibits inflammation and promotes healing by secreting CD206 and IL-10. According to our experimental results, BMSCs promoted macrophages to differentiate towards an M2-like phenotype, which is consistent with the results of previous studies [59, 60]. Moreover, BMSCs stimulated by EMF not only further promoted the polarization of RAW cells towards the M2 phenotype, which benefits tissue regeneration, but also inhibited the inflammatory response. Upregulated genes, such as TGF-β, are transcribed and translated and act on BMSCs through the paracrine pathway to inhibit osteoclast differentiation, thereby contributing to osteogenesis [61, 62]. These pathways that mediate osteogenic differentiation have been verified many times in previous reports and in our research, which further illustrated that EMF promoted the osteogenic potential of BMSCs and boosted the paracrine function of BMSCs to facilitate angiogenesis and osteoimmunomodulation (Fig. 8).

In the in vivo experiments, the repair of cranial defects was not perfect. This result may be related to the inadequate time for animal rearing caused by COVID-19. The fact that there was little new bone formation in the blank group indicated that critical-sized bone defects were difficult to self-heal and that it is necessary to involve biomaterials in treatment. The PCL/HA scaffolds with polydopamine surface modification demonstrated desirable osteoconductive and osseointegration properties during bone repair. However, stem cells on scaffolds can better promote osteogenesis in vivo with the assistance of EMF. In general, EMF can promote multifunctionality of BMSCs to contribute to bone regeneration. The technology of individualized 3D printing ensures anastomosis between scaffolds and defect sites. Advanced materials possess ideal osteoconductive and osseointegration capabilities. Combining the advantages of different technologies to generate a new treatment system is of great significance for clinical treatment.

## Conclusions

In summary, BMSCs stimulated by EMF (15 Hz, 0.3 mT) improve sensitivity to BMP signals by upregulating the quantity of specific BMP receptors. Cascade reactions will be initiated to promote osteogenic differentiation once these stimulated cells receive BMP signals from the surrounding milieu. Furthermore, EMF enhances the paracrine function of BMSCs to facilitate angiogenesis and osteoimmunomodulation. It has been confirmed that the effect of EMF on bone regeneration involves a comprehensive and complex process. The combination of EMF and tissue engineering techniques optimizes the regeneration ability of BMSCs while avoiding the adverse effects of EMF. Our research highlights the profound impact of EMF on tissue engineering and provides an effective strategy for EMF clinical treatment of bone defects.

## Supplementary Information

**Additional file 1: Figure S1.** Fluorescence quantitative analysis of OPN, OCN and Runx2 (*n* = 6). * *P* < 0.05 compared to N-N, ** *P* < 0.01 compared to N-N, ^#^ P < 0.05 compared to O-N, ^##^ P < 0.01 compared to O-N.

**Additional file 2: Figure S2.** Fluorescence quantitative analysis of BMPR1B, BMPR2 and P-Smad1/5/8 (n = 6). * P < 0.05 compared to N-N, ** P < 0.01 compared to N-N, ^#^ P < 0.05 compared to O-N, ^##^ P < 0.01 compared to O-N, ^ P < 0.05 compared to N-EMF, ^^ P < 0.01 compared to N-EMF, ^&^ P < 0.05 compared to O-EMF, ^&&^ P < 0.01 compared to O-EMF.

## Data Availability

The datasets used and/or analysed during the current study are available from the corresponding author on reasonable request.

## Abbreviations

EMFs: Electromagnetic fields
PCL: Polycaprolactone
HA: Hydroxyapatite
BMSCs: Bone marrow mesenchymal stem cells
BTE: Bone tissue engineering
OM: Osteogenic medium
FDM: Fused deposition modeling
FBS: Foetal bovine serum
PBS: Phosphate-buffered saline
SEM: Scanning electron microscopy
CT: Computed tomography
HE: Haematoxylin and eosin
BV: Bone volume
TV: Total volume
BMD: Bone mineral density
ALP: Alkaline phosphatase
BMP2: Bone morphogenetic protein 2
OPN: Osteopontin
BMPR1B: Type IB BMP receptor
BSA: Bone serum albumin
Runx2: Runt-related gene 2
COL1: Type 1 collagen
BMPR1A: Type IA BMP receptor
BMPR1B: Type IB BMP receptor
BMPR2: Type II BMP receptor
ACVR1: Type I activin receptor
