# Supplementary figures and images for: Sinusoidal electromagnetic fields accelerate bone regeneration by boosting the multifunctionality of bone marrow mesenchymal stem cells
Source: Stem Cell Res Ther. 2021 Apr 13;12:234. doi: 10.1186/s13287-021-02302-z (PMC8042357; doi:10.1186/s13287-021-02302-z)

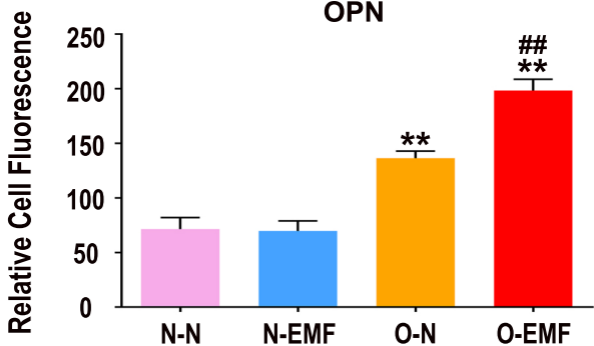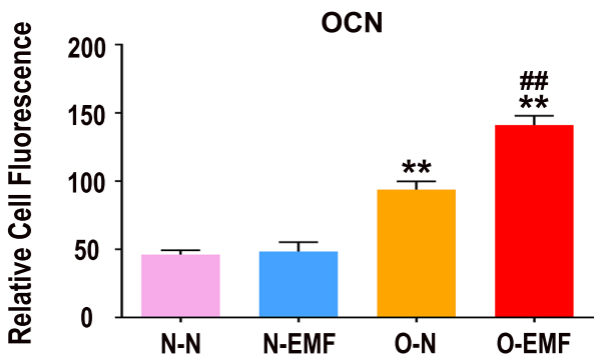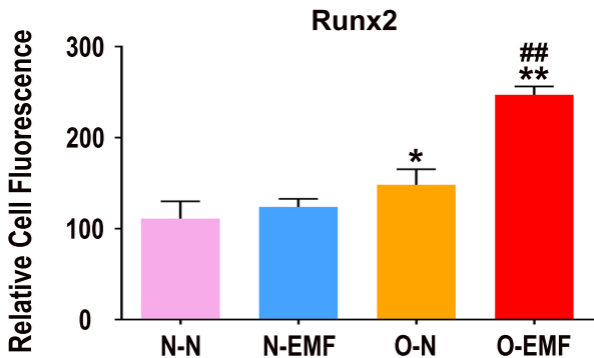

Supplement: Supplementary file 1 — Additional file 1: Figure S1. Fluorescence quantitative analysis of OPN, OCN and Runx2 (n = 6). * P < 0.05 compared to N-N, ** P < 0.01 compared to N-N, # P < 0.05 compared to O-N, ## P < 0.01 compared to O-N. [file 13287_2021_2302_MOESM1_ESM.pdf]

### BMPR1B

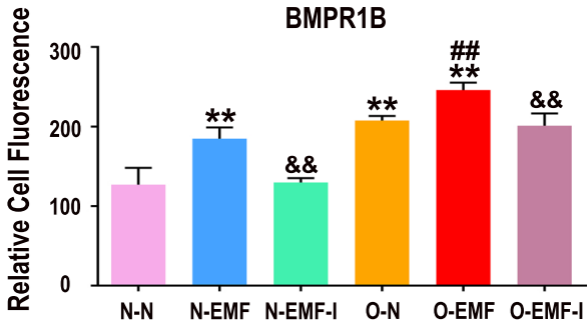

### BMPR2

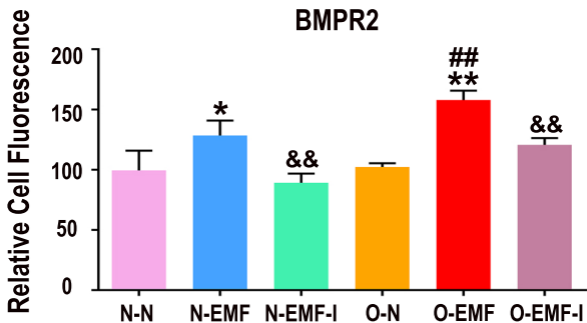

### p-Smad1/5/8

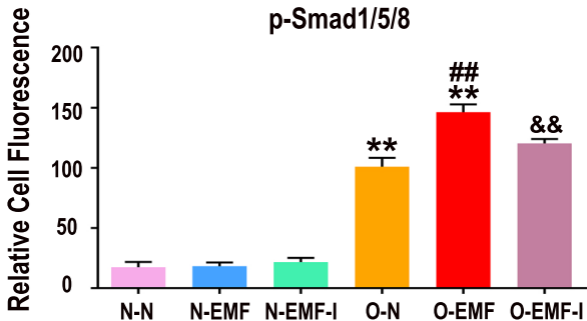

Supplement: Supplementary file 2 — Additional file 2: Figure S2. Fluorescence quantitative analysis of BMPR1B, BMPR2 and P-Smad1/5/8 (n = 6). * P < 0.05 compared to N-N, ** P < 0.01 compared to N-N, # P < 0.05 compared to O-N, ## P < 0.01 compared to O-N, ^ P < 0.05 compared to N-EMF, ^^ P < 0.01 compared to N-EMF, & P < 0.05 compared to O-EMF, && P < 0.01 compared to O-EMF. [file 13287_2021_2302_MOESM2_ESM.pdf]
